# Pathophysiology of Cardiovascular Diseases: New Insights into Molecular Mechanisms of Atherosclerosis, Arterial Hypertension, and Coronary Artery Disease

**DOI:** 10.3390/biomedicines10081938

**Published:** 2022-08-10

**Authors:** Weronika Frąk, Armanda Wojtasińska, Wiktoria Lisińska, Ewelina Młynarska, Beata Franczyk, Jacek Rysz

**Affiliations:** Department of Nephrology, Hypertension and Family Medicine, Medical University of Lodz, ul. Zeromskiego 113, 90-549 Lodz, Poland

**Keywords:** cardiovascular disease, atherosclerosis, arterial hypertension, coronary artery disease, inflammation, matrix metalloproteinases, oxidative stress, vascular endothelium dysfunction, genetic factor

## Abstract

Cardiovascular diseases (CVDs) are disorders associated with the heart and circulatory system. Atherosclerosis is its major underlying cause. CVDs are chronic and can remain hidden for a long time. Moreover, CVDs are the leading cause of global morbidity and mortality, thus creating a major public health concern. This review summarizes the available information on the pathophysiological implications of CVDs, focusing on coronary artery disease along with atherosclerosis as its major cause and arterial hypertension. We discuss the endothelium dysfunction, inflammatory factors, and oxidation associated with atherosclerosis. Mechanisms such as dysfunction of the endothelium and inflammation, which have been identified as critical pathways for development of coronary artery disease, have become easier to diagnose in recent years. Relatively recently, evidence has been found indicating that interactions of the molecular and cellular elements such as matrix metalloproteinases, elements of the immune system, and oxidative stress are involved in the pathophysiology of arterial hypertension. Many studies have revealed several important inflammatory and genetic risk factors associated with CVDs. However, further investigation is crucial to improve our knowledge of CVDs progression and, more importantly, accelerate basic research to improve our understanding of the mechanism of pathophysiology.

## 1. Introduction

Cardiovascular diseases (CVDs) are a group of disorders of the heart and blood vessels [1]. They are a set of heterogeneous diseases whose underlying cause of development is most often atherosclerosis [2]. CVDs are chronic diseases that gradually evolve throughout life and remain asymptomatic for a long time [3]. Moreover, CVDs are the leading cause of morbidity and mortality in patients worldwide [4]. In Europe, CVDs are responsible for 45% of deaths [5], thus being particularly important for public health. Atherosclerosis, coronary artery disease (CAD), and arterial hypertension (AH) are the leading causes of CVDs [6].

Atherosclerosis is the main cause of cardiovascular-related death worldwide [7]. It is a thickening and hardening of the arterial wall, accompanies aging, and is related to major adverse impact on the cardiovascular system and various other diseases [8]. Elevated plasma cholesterol level (>150 mg/dL) is a major cause of the development of atherosclerosis [9].

CAD is a common heart condition in which we can observe the narrowing or blockage of major blood vessels—coronary arteries. CAD is caused primarily by plaque formation within the intima of the vessel wall [10], with plaque being defined as a fatty material growing inside intima along with a severe inflammation, especially if the inflammation is chronic. This in turn causes difficulties in supplying the cardiomyocytes with enough blood, oxygen, and nutrients [11]. As a result, atherosclerotic plaque may erode or rupture, initially resulting in thrombosis and then a closure of the vessel, leading to myocardial infarction, stroke, limb ischemia, and death [12]. The other factors causing this condition are a diseased endothelium, low-grade inflammation, and lipid accumulation [13].

AH is one of the most common CVDs. AH causes few or no symptoms, but is an important risk factor for a myocardial infarction, stroke, renal failure, and peripheral vascular disease [14]. The diagnosis of AH, in accordance with the most significant guidelines, is diagnosed when a person’s systolic blood pressure (SBP) in the office or clinic is ≥140 mm Hg and/or their diastolic blood pressure (DBP) is ≥90 mm Hg following a repeated examination [15]. CVDs are caused by multiple factors. Some of them are unvarying, such as age, gender, and genetic background, whereas others could be variable and, therefore, reduced (smoking, physical inactivity, poor dietary habits, elevated BP, type 2 diabetes, dyslipidemia, and obesity) [16].

In this review, we summarize the available evidence of the pathophysiological implications of CVDs, focusing on atherosclerosis, CAD, and AH. It is particularly essential to explain the mechanisms of their formation and progression.

## 2. Coronary Artery Disease and Atherosclerosis

CAD and atherosclerosis are discussed first, due to their broad subject matter and mutual implication. The pathophysiological basis of these diseases is constantly being researched in order to finally find the reasons behind their formation and what factors additionally exacerbate the ongoing processes and may directly contribute to their induction. Understanding the exact course of the entire pathophysiological and pathogenic process will allow for accurate prevention and diagnosis of both diseases. Cardiovascular diseases are classified as civilization diseases [17]; hence, it is important to create accurate and effective algorithms that will help doctors during their work. Those that already exist require further improvement and improvement, due to the continuous discoveries and introduction of new pharmacotherapeutic solutions.

In our review, we focus on endothelial dysfunction as one of the first and most important causes of the processes leading to CAD and atherosclerosis [18]. In the case of both diseases, these processes constitute a starting point for further research and implications in the course of the disease development. This brings us to the remainder of the paper, i.e., inflammatory processes involving diseased tissue and oxidative factors.

We also do not forget about the genetic basis; dynamically developing research shows completely new faces of these diseases known to us and makes it possible to reflect on the real causes of their diversity and changeable forms, as well as create new possibilities for better diagnostics and considering whether the current judgement that the lifestyle and drugs mainly allow controlling the disease-causing process. They allow the use of new solutions and deepen our knowledge in this subject.

### 2.1. Endothelial Dysfunction in Atherosclerosis

In arterial vasculature there are areas (branch points, bifurcations, and major curvatures—arterial geometry), which are much more prone to atherosclerotic lesions [19]. The mechanical forces such as a turbulent flow, which is related to the geometry and shape of vessels, also influence the endothelial cell [19].

Atherosclerosis occurs in these regions as a result of differences in flow, which is present at sites of low shear stress, turbulence, and oscillating flow. We do not maintain that these factors cause atherosclerosis but rather that they “prime the soil” in which lesions start to develop [20]. Endothelial cells are exposed to various degrees and types of shear stress, which have influence on their shape, intracellular signaling, and gene expression [21]. In the regular state, which is the quiescent state of the endothelium, nitric oxide (NO) is produced in order to bind to cysteine groups in NF-κB and the mitochondria, which inhibit cellular processes. Moreover, the endothelial layer is covered by a glycocalyx, which is layer of proteoglycans and extracellular matrix components, involved in transendothelial transport, e.g., of lipoproteins, which can be lost or reduced in inflammation due to plasminogen activator inhibitor [21]. In homeostasis, endothelial cells prevent platelet activation, blood clotting, and leukocyte adherence by secreting substances such as NO, prostacyclin, t-PA, and antithrombin III [21]. When inflammation occurs there is an increase in the number of adhesion molecules (E-selectin, ICAM, and VCAM), which participate in the infiltration of leukocytes through the endothelial layer [22]. With leukocytes, lipoproteins penetrate the endothelium, and they are trapped in the subendothelial space and oxidatively modified. Endothelial dysfunction in modern cardiovascular medicine is described as changes in the production and availability of endothelial-derived NO, prostacyclin, and endothelin, as well as their impact on vascular reactivity. In this case, reactive oxygen species (ROS) such as H_2_O_2_ reach the regulatory molecules, which leads to the activation of the cells [23]. The endothelial membrane is permeable to compounds such as NO and H_2_O_2_, which causes the activation of the transcription factors and protease. Moreover, production of endothelial ROS may be triggered by inflammation and cells that participate in this process, such as leukocytes and growth factors. Other mechanisms that cause endothelial dysfunction are the formation of peroxynitrite, NO synthase uncoupling, prostacyclin formation inhibition, endothelin expression stimulation, and reduced NO signaling due to the inhibition of soluble guanylate cyclase activity [24]. All these mechanisms promote a vasoconstrictive and procoagulant milieu. Moreover, endothelial cells can make a transition to the mesenchymal cells [21]. Consequently, the extracellular matrix deposits between cells and dysregulates the junctional proteins (e.g., occludin and claudin-5), which lose cell–cell contact [21]. As a result, the endothelium loses its integrity with media, and this causes a higher activity in that field. Then, the changes extend beyond NO metabolism reactivity, including increased level of permeability for lipoprotein, oxydation, leukocyte adhesion and accumulation, and altered extracellular matrix metabolism, with all of these accumulating in the arterial wall [19]. In this way, macrophages, cholesterol, and inflammatory cells access the media, and atherosclerosis begins. Moreover, the mechanical forces such as a turbulent flow, which is related to the geometry and shape of vessels, also influence the endothelial cell [19].

### 2.2. Inflammatory and Oxidising Factors in Atherosclerosis

Following the mechanisms of endothelial dysfunction, we can see that inflammatory factors also play a huge role in the development of this pathology. Atherosclerosis is characterized by the retention of lipids and inflammatory cells such as macrophages, T lymphocytes, and mast cells in damaged arterial wall, the intima [25]. Modified lipids activate inflammatory cells in the intima, producing chemokines and cytokines such as tumor necrosis factor (TNF-alpha), interleukin -1, -4, and -6, and interferon-gamma, which activate other leukocytes, endothelial cells, and adhesion molecules, especially vascular cell adhesion molecule-1 (VCAM), intercellular adhesion molecule-1 (ICAM), and E-selectin, on the endothelial surface. These, in turn, recruit other inflammatory cells. As a result, the monocyte-derived macrophages release enzymes in order to modify the lipoproteins. These modified lipoproteins become atherosclerotic plaques. Then, macrophages absorb and build in the cholesterol-rich lipoproteins from LDL, as well as secrete pro-oxidant substances, which contribute to the process of atherosclerosis: ROS and RNS (reactive nitrogen species). These are the same compounds that participate in the endothelial dysfunction, which aggravates the condition of the endothelium, indicating that the process is self-perpetuating. This is not desirable, because this damage of the cellular functions of biomolecules (such as proteins, carbohydrates, and lipids) can result in lipid peroxidation and LDL oxidation. As we know, oxidized phospholipids trigger inflammation, because of the extensive binding to the Toll-like receptors, which can activate the transcription factors nuclear factor-κB (NF-κB) cytokines which trigger proinflammation; hence, oxLDL is called a clinical marker of plaque inflammation [26]. OxLDL irritates endothelial cells, increasing the production of adhesion molecules. ROS and RNS convert the LDL-C to the OX-LDL, which are built into the intimal layer. The inner layer also migrates the muscle cells from the media and proliferates itself. When all these processes combine, the atherosclerotic plaque is created, featuring fiber tissue, muscle cells, and many inflammatory cells. Accelerated cell turnover is likely to lead to an enhanced macromolecular permeability, increasing lipid uptake in the regions with a disturbed flow. This in turn would lead to the atherosclerotic phenotype expression of VEGF increasing in response to low shear stress, leading to greater endothelial permeability [27]. In addition, in the vessels, hyperglycemia promotes the overproduction of ROS by the mitochondrial electron transport chain. Excess superoxide leads to DNA strand breakage and activation of nuclear poly ADP ribose polymerase (PARP). These processes inhibit glyceraldehyde-3-phosphate dehydrogenase (GAPDH), shunting the early glycolytic intermediates into the pathogenic signaling pathways during inflammation [28].

### 2.3. Epigenetic Factors in Atherosclerosis

The first major epigenetic mechanism that contributes to the complexity of atherosclerosis is DNA methylation, which is catalyzed by DNA methyltransferase 1 (DNMT1) and 3b (DNMT3b) DNA methylation [27]. Moreover, DNA demethylation is also an important mechanism explaining the pathogenesis of atherosclerosis. During studies on mice, the *TET2* overexpression significantly reduced atherosclerotic lesion formation, likely by oxidatively demethylating 5meC to 5hmC in the endothelial vessel wall. This *TET2*-induced rescue occurs via the upregulation of autophagy, as *TET2* overexpression decreases the methylation level of the promoters of autophagic flux-related genes [29]. Moreover, the loss of *TET2* functions in hematopoietic cells and myeloid cells enhanced atherosclerosis in mice, as shown in [30]. Bone marrow was transplanted from control mice into an atherosclerosis-prone *Ldlr−/−* recipient mice, and a diet high in cholesterol was introduced. After 5, 9, and 13 weeks on the diet, the recipients of *Tet2−/−* marrow had 2.0-fold, 1.7-fold, and 1.4-fold larger lesions in the arteries. DNA methylation and histone acetylation are key processes in regulating the expression of inflammatory cytokines and chemokines in atherosclerosis. This involves methylation at the C5 position of cytosine residues in a *CpG* dinucleotide context, exerted by DNA methyltransferases (DNMTs). DNMTs are capable of both methylation and demethylation, making the modification reversible, but those modifications are reserved for the Tet methylcytosine dioxygenases (TET1, 2, and 3) [29]. In addition, studies have shown that other genes, *DNMT3A*, *JAK2*, and *ASXL1*, which mutated, increase the risk of incident coronary heart disease 12-fold in *JAK2* V617F and 1.7-fold to 2.0-fold in other genes mentioned above [30]. Mutations in the *DNMT3A* and *TET2* influence DNA methylation, whereas those in the *ASXL1* alter histone modifications, thereby influencing clonal expansion of hematopoietic stem cells [31]. This phenomenon was found to influence risk of CAD [32].

### 2.4. Endothelial Dysfunction in CAD

The vascular endothelium is the layer of cells lying under the epithelium lining the inside of the vessel and the muscular layer, which is a boundary between the circulating blood and the vascular wall. Its cells are specialized in maintaining vascular homeostasis, which is crucial for the proper functioning of organs, especially the heart. Through its role in signal transduction and as a source of many vasoactive substances, it is their key regulator. The vascular endothelium reacts to physical and chemical stimuli through the release of autocrine and paracrine vasoactive agents. Factors of endothelial origin regulate surface tension and cell adhesion, including platelet activation and leukocyte adhesion, smooth muscle cell proliferation, and vascular wall inflammation. The endothelium is considered to be a strong indicator of cardiovascular function and fitness. Its dysfunction is considered to be the earliest marker of atherosclerosis and, in effect, CAD.

First, we focus on the critical process of the NO signaling pathway [33]. Nitric oxide is a gas with relatively small particles that strongly dilates blood vessels and has additional anti-inflammatory and antioxidant properties [34]. It is synthesized by three distinct subtypes of the NO synthase (NOS) enzyme, each with unique expression patterns and functional properties: neuronal NOS (nNOS, NOS1), inducible NOS (iNOS, NOS2), and endothelial NOS (eNOS, NOS3). Broadly, these proteins catalyze the production of NO and l-citrulline from l-arginine and O_2_, using electrons donated from dihydronicotinamide adenine dinucleotide phosphate (NADPH). Finally, in the presence of heme and tetrahydrobiopterin (BH_4_), NOS monomers form homodimers, which are capable of using the donated NADPH electrons to catalyze the two-step oxidation of l-arginine to l-citrulline and NO. The expression of the nicotinamide adenine dinucleotide phosphate oxidase in the vessel wall with the consequent overproduction of NO has been proposed as an initial step in the chronic dysregulation of normal NO production by eNOS, which is characteristic of the monomeric forms of eNOS. In effect, eNOS produces a superoxide, rather than NO. The superoxide reacts with NO to form peroxynitrite, which in turn increases the uncoupling of eNOS and further superoxide production [35,36]. This again promotes eNOS uncoupling and is itself a mediator of effects [37]. When uncoupled, eNOS switches from its oxygenase-induced oxidant excess, and then exerts a deleterious effect on the endothelial and vascular function.

In the second step, NOS catalyzes the oxidation of *N*^ω^-hydroxy-l-arginine to l-citrulline, thereby releasing NO [38,39]. For example, nNOS and eNOS are highly dependent on Ca^2+^-activated CaM for homodimerization and activity, whereas iNOS is minimally dependent on calcium concentration. These nuances have critical functional effects. NADPH oxidase (NOX) is another element that has received much attention as a key element in the development of vascular dysfunction. NOX has the primary function of producing reactive oxygen species (ROS) and is considered the main source of the ROS production in endothelial cells. The enzymatic production of NO by eNOS is critical in mediating the endothelial function, and oxidative stress can cause eNOS dysregulation and endothelial dysfunction [40].

Taking this into account, the endothelial dysfunction is directly related to a decreased production and sensitivity of cells to NO. As a result, we have an effective disturbance in the functioning of the entire vessel and its homeostasis, which leads to an observation of prothrombotic and proinflammatory phenomena, along with lower susceptibility of the blood vessel wall. 

**Figure 1:**
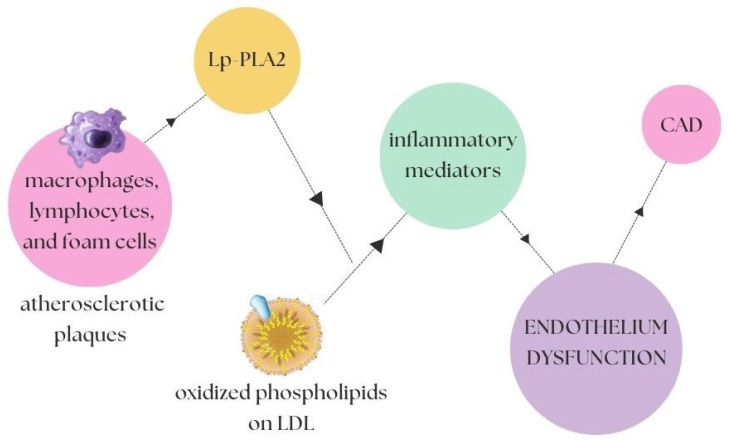
Lp-PLA2-dependent activation cycle [40]. The macrophages, lymphocytes, and foam cells present in the atherosclerotic plaques have an influence on the increased level of Lp-PLA2, which in turn catalyzes a reaction that, in the presence of oxidized phospholipids on LDL, is a direct contribution to the secretion of an increased amount of inflammatory mediators, which in turn leads to endothelial dysfunction and further CAD.

Another factor of interest is phospholipase A2 and its influence on the endothelial dysfunction. Lp-PLA2 (lipoprotein-bound phospholipase A2), also known as platelet-activating acetylhydrolase, is a vascular-specific inflammatory enzyme mainly produced by macrophages, lymphocytes, and foam cells in the atherosclerotic plaques. The circulation of Lp-PLA2 is mainly associated with apolipoprotein B-containing lipoproteins and, therefore, closely related to low-density lipoproteins (LDLs). Lp-PLA2 can trigger proinflammatory and proatherogenic properties in the vascular wall. The enzyme hydrolyzes oxidized phospholipids on the LDL particles in the intima of the artery, producing two highly inflammatory mediators with proinflammatory and atherosclerotic effects. Elevated levels of Lp-PLA2 correlate with arterial stiffness in patients with a stable CAD (Figure 1), regardless of the risk factors and pharmacotherapy [41].

### 2.5. Inflammation in CAD

Systemic vasculitis is a term referring to a group of diseases characterized by inflammation and fibrinoid necrosis of blood vessel walls. The underlying pathogenesis involves many mechanisms such as cell-mediated inflammation, immune complex (IC)-mediated inflammation, and ANCA-mediated inflammation, For example, inflammation in GCA is mostly a T-cell-driven process in which dendritic cells present antigens in blood vessel walls. These T-cells activate other inflammatory cells such as monocytes and macrophages, which, as a result, release proinflammatory cytokines (interleukin-1, interleukin-6, and interferon-γ). Inflammation in diseases such as polyarteritis nodosa and cryoglobulinemia is driven by IC deposition (antibody-mediated IC formation, microaneurysms) [42], whereas Wegener’s granulomatosis, Churg–Strauss syndrome, microscopic polyangiitis, and necrotizing glomerulonephritis result from interactions between antibodies and enzymes within inflammatory cells, which is typical for ANCA-related vasculitis [43]. As a consequence of these processes, manufactured antibodies and immune complexes attach to the inner layer of blood vessels. This is a cause of aggravated ET-1 release, which, in a positive feedback loop, recruits more monocytes and macrophages. Vasculitis, as a very diverse group of diseases, has many cellular mechanisms that we mentioned above and affects different arteries and veins, in terms of both distribution and size [44]. The size of vessels is also crucial in terms of factors sustaining inflammation. Vasculitis of small veins is related to involvement of the endothelium, necrose (which is associated with ANCA), damage of the arterial walls, aneurysm formation, and hemorrhage. It is also characterized by leukocytoclasia, which is the excessive accumulation of neutrophils. Moreover, in vasculitis of large systemic vessels, the response to the inflammation leads to thickening of the intima, restenosis, and overall remodeling. These changes are manifested in the malfunctioning of blood vessels and subsequent events such as infarction and hemorrhage [44].

### 2.6. Genetic Background in CAD

In recent years, there has been substantial research showing correlations between genetic factors and endothelial function and dysfunction, which in turn are associated with an increased risk of developing CAD. In our work, we selected genes that, in our opinion, have gained attention in recent years and have shown a significant impact on the development of CAD. However, it should be remembered that, since 2007, in addition to all the genes known so far that have a confirmed impact on CAD, new research has resulted in nearly 60 distinct genetic loci [45].

As mentioned before, atherosclerosis underlies CAD. Previous studies have shown that it has an important but poorly understood and defined genetic component [41,46]. An earlier genome-wide association study (GWAS) identified many loci associated with an increased risk of CAD. A recent study by Redouane Aherrahrou, Liang Guo, and others accurately described the characteristics of atherosclerosis that are associated with the migration and proliferation processes in the vascular smooth muscle (VSMC) cells. The phenotypic variability shown was of great importance here; more specifically, four loci directly related to the atherosclerosis in VSMC were identified. Moreover, as many as 79 out of 163 loci associated with CAD are associated with one of the VSMC phenotypes [47].

One of them is the chromosome 1q41 locus, which harbors *MIA3* protein. The G allele of the lead risk SNP rs67180937 is associated with a lower VSMC *MIA3* expression and a lower proliferation. The *MIA3* protein, which plays a role in the secretion of collagen, is the likely cause of the genetic basis of CAD at its 1q141 locus. Silencing this protein resulted in a reduction in the VSMC profiling, with a lentivirus used for this purpose. This protein also significantly impacts the thickness of the fibrous cap of the atherosclerotic plaque, both in humans and in mice [48].

Next one, *JCAD* (junctional cadherin 5-associated, also known as *KIAA1462*, encoding a junctional protein associated with CAD) is one of more than 160 GWAS-identified genes [49]. *JCAD* is a protein that binds cells in the endothelium and is responsible for the regulation of pathological angiogenesis, less frequently than its development [50]. CAD depletion also increased EC apoptosis and reduced EC proliferation, migration, and angiogenesis [51]. *JCAD* depletion inhibits the activation of the YAP/TAZ pathway and the expression of further proatherogenic genes including CTGF and Cyr61. Proteomic studies suggest that *JCAD* regulates the YAP/TAZ activation by interacting with the actin-binding protein TRIOBP, thus stabilizing stress fiber formation. In addition, endothelial *JCAD* expression was increased in murine and human atherosclerotic plaques [52]. 

**Figure 2:**
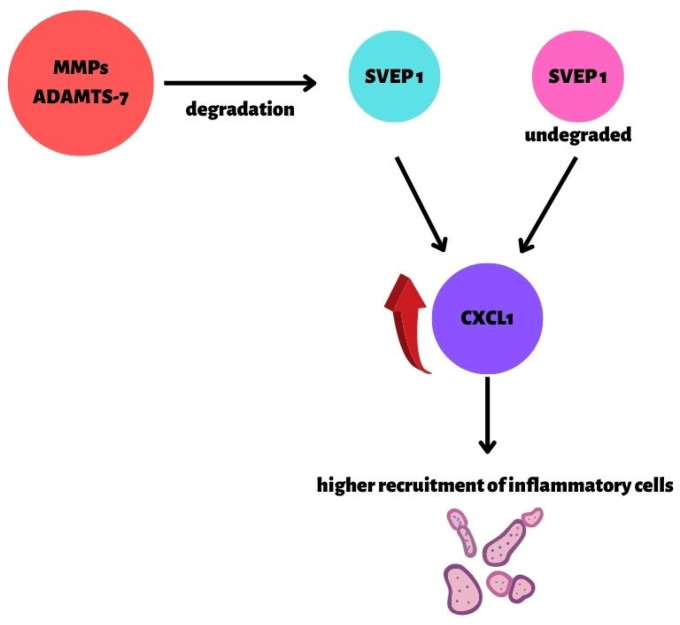
The role of *SVEP1* in atherosclerosis and CAD [56]. *CXCL1* expression is dependent on the presence of *SVEP1*. Only in the presence of *SVEP1* is the *CXCL1* expression silenced. Two factors have a significant influence on the expression of *CXCL1*: MMPs and ADAMTS- 7 (specific metalloproteinases), which can reduce the wildtype *SVEP1*; mutant *SVEP1* (*SVEP1_p.D2702G* missense variant). Consequently, the secretion and recruitment of inflammatory cells are increased.

Moreover, the SIRT1 protein (Sirtuin 1) plays an important role in regulating the physiological mechanisms taking place in the cell, consequently influencing the mechanisms against CAD. SIRT1 is a cardioprotective molecule due to it regulating the expression of eNOS. It regulates angiogenesis, and it protects the endothelium against dysfunctional changes and damage to the heart muscle resulting from a reduced perfusion and ischemia. Suppression of the SIRT1 causes monocyte affinity due to endothelial dysfunction [53]. The gene encoding transcription factor *TCF21* has been linked to CAD risk by GWAS in multiple racial/ethnic groups. *TCF21* antagonizes the MYOCD/SRF pathway through multiple mechanisms, further establishing a role for this CAD-associated gene in smooth muscle cells [54]. Two genes associated with high susceptibility of atherosclerotic plaque were also found. These two genes changed at different timepoints after myocardial infarction, and both had the lowest prognosis of heart failure when expressed at low levels. *TLR2* and *CD14* are closely associated with the worsening of CAD, the instability of atherosclerotic plaques, and the prognosis of heart failure after myocardial infarction [55]. Other studies revealed an atheroprotective role of *SVEP1*. The deficiency of wildtype *SVEP1* increased the endothelial *CXCL1* expression, leading to an enhanced recruitment of proinflammatory leukocytes from blood to plaque. Consequently, elevated vascular inflammation resulted in an enhanced plaque progression in the *SVEP1* deficiency (Figure 2) [56]. An intronic region of the disintegrin and metalloproteinase with thrombospondin motifs-7 (*ADAMTS7*) is one of the two crucial factors that can reduce the wildtype *SVEP1* [57]. *ADAMTS7* is one of the many proteins involved in the remodeling of the blood vessel walls due to the properties that dissolve their substrate proteins [57]. Increased expression of this gene is associated with an increased proteolytic activity and a migration of smooth muscle cells, which favors the abovementioned remodeling properties, as demonstrated on the smooth muscle cells in mice [58].

Two more genes have attracted our attention: *NOS3* and *GUCY1A3* and their common variants. Their presence significantly contributes to a reduction in the level of BP, which is known to be one of the important factors in reducing the occurrence of CAD [59]. Loss-of-function mutations in GUCY1A3 and CCT7 result in a reduced level of α1 subunit of soluble guanylyl cyclase (α1-sGC), as well as β1-sGC protein content, and they impair the soluble guanylyl cyclase activity, which was correlated with risk of myocardial infarction in a large family enriched for premature CAD^2^ [60]. 

The following genes, when inactivated, show increased risk of CAD: *LDRL* (low-density lipoprotein receptor), *APOA5* (apolipoprotein A-V) [61], and *LPL* (lipoprotein lipase) [45]. Abnormal mutations in *APOA5* increase the risk of CAD due to this gene coding a protein responsible for the increased activity of *LPL* [61]. The opposite effect is shown by mutations in the *APOC3* and *ANGPTL4* genes, which are responsible for the inhibition of lipoprotein lipase [62,63].

## 3. Arterial Hypertension

### 3.1. Matrix Metalloproteinases in AH

**Figure 3:**
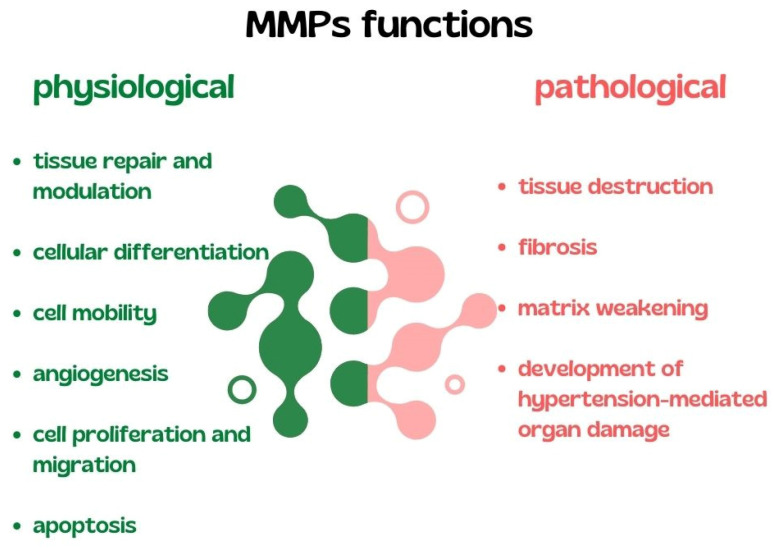
Summary of physiological and pathological functions of MMPs. MMPs, matrix metalloproteinases.

The remodeling of the vascular extracellular matrix (ECM) during hypertensive impairment has been associated with the involvement of matrix metalloproteinases (MMPs) and their tissue inhibitors (TIMPs). Several MMPs and TIMPs might participate in the vascular remodeling associated with the AH. Matrix metalloproteinases (MMPs) are a family of zinc-dependent endopeptidases, involved in many physiological and pathological processes, particularly tissue repair and modulation, cellular differentiation, cell mobility, angiogenesis, and cell proliferation, migration, and apoptosis. The dysfunction of MMP activity leads to a progression of various pathologies, tissue destruction, fibrosis, and matrix weakening [64]. It is worth mentioning that MMPs are also involved in the development of hypertension-mediated damage to the vascularity, heart, and kidneys, leading to organ failure and cardiovascular complications (Figure 3) [65]. Endogenous tissue inhibitors of MMPs (TIMPs) are crucial in restraining the degradation of ECM. Furthermore, the pathological processes in the vessel wall might be provoked by an excessive amount of MMPs, due to an imbalance between MMPs and TIMPs [66].

The results of a meta-analysis conducted by Marchesi et al. showed increased plasma levels and activities of MMP-2, MMP-9, and TIMP-1 among hypertensive patients [67]. Furthermore, Bisogni et al. found that a high BP along with the development and progression of CVDs correlated with increased levels of MMP-2 and MMP-9. It is also worth mentioning that MMP-9 is involved in the vascular remodeling, thus leading to the perpetuation of the elevated BP [65]. Additionally, Kostov et al. reported elevated MMP-1 levels and increased collagen degradation in patients with AH [68]. Thus, the impaired vascular remodeling and aggravated proteolytic activity might result from an MMP/TIMP imbalance in vascularity, principally in the intima and media of the vessel wall [16]. On the contrary, a study by Basu et al. attempted to demonstrate a crucial role of the TIMP-3, which is the protection of the arterial ECM in response to Ang II [69]. Furthermore, Pushpakumar et al. observed worsening of kidney functions and renovascular remodeling due to an increased activity of MMP-9 among hypertensive mice with TIMP2 deficiency [70]. These alterations in clinical AH suggest an important role for MMPs in AH. What is interesting, the determination of the MMPs and TIMPs in the serum can be used as a noninvasive approach to diagnosing and monitoring the structural changes in the cardiovascular system in AH [71].

Alterations in the structure, along with the degradation of several components of the vascular wall, including ECM, are critical during AH. The ECM is critical for maintaining the homeostasis in vasculature. It is thought to support stability and vascular cell behaviors [72]. A study by Cui et al. indicated that MMPs cause a degradation of the ECM proteins such as collagen or elastin; therefore, MMPs disturb the structural integrity of the vascular wall and lead to a decreased elasticity of the vascular wall [73].

Vascular ECM remodeling during AH applies effects on the functional and structural alterations in the vascular smooth muscle cells (VSMCs). Wang et al. demonstrated that MMPs promote VSMC growth and proliferation by activating growth factors such as insulin growth factor-1, transforming growth factor-h, and heparin-bound epidermal growth factor, in addition to promoting interactions between VSMC and these growth factors [16].

**Figure 4:**
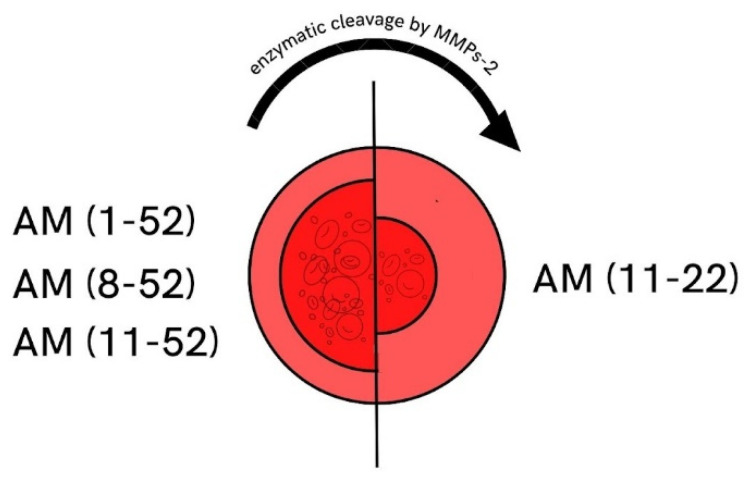
MMP-2 cleavage of AM. Adrenomedullin (AM 11–52) is a peptide that affects vessels and leads to its vasodilation. MMP-2 regulates BP by reducing the AM (1–52, 8–52, and 11–52) into smaller peptides AM (11–22), which act as vasoconstrictors. Therefore, MMP-2 may worsen vascular function and increase BP in hypertensive patients. Numbers in brackets are the peptide length before and after the MMP2 cleavage. MMP-2, matrix metalloproteinase 2; AM, adrenomedullin; BP, blood pressure.

Martínez et al. found that, in rats, MMP-2 may regulate BP by destroying the vasodilator peptides adrenomedullin (AM) 1–52, 8–52, and 11–52 (Figure 4). AM plays a role in the regulation of BP. MMP-2 cleaves the vasodilator peptide AM (1–52, 8–52, and 11–52) into smaller peptides AM (11–22), which act as the vasoconstrictors. MMP-2 activity may be related to the development of AH, both by reducing the levels of the potent vasodilator AM (1–52, 8–52, and 11–52) and by generating hypertensive molecules [74].

In addition, the MMPs may play a role in hypertensive complications such as intracranial hemorrhage. A study by Wakisaka et al. found increased levels of MMP-9 in the endothelial cells and ECM of cerebral vessels that are considered to be associated with a spontaneous intracranial hemorrhage in the hypertensive rat models [75].

MMPs/TIMPs are involved in the regulation of the ECM metabolism, which plays a significant role with regard to the maintenance of tissue integrity [72].

### 3.2. Immune System in AH

Both innate and adaptive immune cells have been implicated in the development of AH. The majority of studies correlated the presence of AH with elevated levels of circulating inflammatory markers, cytokines, and antibodies. The findings by Mirhafez et al. showed an association between the concentrations of several cytokines and AH (an increased level of IL-6, IL-1β, IL-1α, IL-18, IL-2, IL-8, TNF-α, IFN-γ, C-reactive protein (CRP), and MCP-1, and a decreased level of IL-10) among patients with AH [76].

Sesso et al. evaluated the relationship between IL-6 and CRP and the risk of developing AH in a nested case–control study of 400 women. The results showed that IL-6 was indifferently associated with while CRP was firmly related to the AH risk [77]. Furthermore, CRP has been found to be a mediator in the development of endothelial dysfunction, vascular stiffness, and elevated BP [78]. Kong et al. investigated CRP gene polymorphisms. The results showed that plasma levels of CRP could predict the development of AH. On the contrary, the relationship between genotype and CRP levels was not associated with a change in AH risk [79]. Lima et al. demonstrated that IL-10 inhibits the pressor activity of Ang II and vascular dysfunction associated with AH, while also modulating the RhoA/Rho kinase pathway. Strategies to increase IL-10 levels during AH may enhance the benefits provided by regular treatments [80]. Peng et al. established in mice models that elevated levels of IL-4 are linked to the development of with cardiomyopathy, as a result of an angiotensin II-induced cardiac damage [81].

Macrophages are the main effector cells of the innate immune system, and numerous studies indicate their role in the pathogenesis of AH. Monocyte levels are elevated in hypertensives in comparison to normotensives [82]. Interestingly Ang II-preactivated circulating monocytes in hypertensive patients might lead to subendothelial infiltration and thereafter augment the risk of arteriosclerotic complications [83].

Macrophages have a role in mediating hypertensive end-organ damage. In one study, perivascular macrophages were associated with the neurovascular and cognitive dysfunction induced by AH [84]. Shen et al. demonstrated that microglia, which are the resident immune cells in the brain, are the main molecular factors in mediating a neuroinflammation and modulating neuronal excitation, which contributes to an increased BP [85].

Barbaro et al. identified a novel mechanism via which excess sodium contributes to inflammation and AH. The result showed a pathway of sodium entrance into dendritic cells. Therefore, high-salt-treated DCs produce the prohypertensive cytokines IL-17 and INF-γ [86].

Antigen-presenting cells (APCs) are involved in the evolution of the inflammation associated with AH. Hevia et al. showed that APCs are essential for the development of AH, as the deletion of APCs produces rapid changes in the BP in mice with angiotensin II plus a high-salt diet. Additionally, the APCs activate the intrarenal renin/angiotensin system components and take part in the modulation of the natriuresis and tubular sodium transporters [87].

The NOD-like receptor protein 3 (NLRP3) inflammasome participates in the development of AH [88]. Krishnan et al. investigated MCC950—a recently-identified inhibitor of NLRP3 activity. The result showed that, in mice with established AH, MCC950 lowered BP and decreased renal inflammation, together with reduced fibrosis and kidney dysfunction [89]. 

Some studies investigated the neuro-immune axis associated with AH. Abboud et al. indicated that excessive sympathetic activity and reduced parasympathetic activity have a major role in pathological processes [90]. Furthermore, Harwani et al. demonstrated anti-inflammatory nicotinic/cholinergic modulation of the innate immune system in rats. Interestingly, they revealed proinflammatory innate immune responses in the hypertensive rats, prior to the development of AH [91].

Evidence from human and animal studies strongly suggests an association between inflammation and AH. Most recent findings in this field add to the growing body of evidence suggesting that AH is an inflammatory disease, while also drawing attention to the new possibilities for treating AH.

### 3.3. Oxidative Stress in AH

Disruption of redox signaling is a common pathophysiological mechanism observed in AH. Oxidative stress, resulting in either enhanced ROS production or decreases in antioxidant defense, is associated with an elevated BP, endothelial dysfunction, and vascular remodeling [92]. Oxidative stress and inflammatory responses act cooperatively in the pathogenesis of AH [93]. In the vascular system, the major source of ROS production is NOX, whose expression is increased in hypertensive conditions [94]. Dysregulation of enzymes such as NADPHNOX, nitric oxide synthase (NOS), xanthine oxidase, mitochondrial enzymes, or superoxide dismutase (SOD), which generate ·O_2_^−^, H_2_O_2_, and ·OH together with reduced levels of antioxidants, results in increased formation of ROS within the vasculature. ROS contribute to vascular injury by promoting VSMC growth, extracellular matrix protein deposition, activation of matrix MMPs, inflammation, endothelial dysfunction, and increased vascular tone [95]. Furthermore, Crowley et al. indicated a novel mechanism via which oxidative stress promotes an inflammation in the vascular wall. The chaperone protein cyclophilin A (CypA), secreted from VSMCs due to ROS stimulation, leads to the recruitment of the inflammatory cells within the vasculature. Additionally, CypA triggers activation of MMPs, thus exaggerating vascular injury [93].

A variety of studies have indicated the role of NOX enzymes in the vascular remodeling during AH. Dikalova et al. indicated that an overexpression in the vascular smooth muscle of NOX1 exacerbates the hypertrophic and hypertensive responses to Ang II and increases the superoxide production in mice. Therefore, NOX1 participates in the development of cardiovascular pathologies [96]. Interestingly, Nosalski et al. investigated the pharmacological inhibition of NOX1/NOX4 in rats. The result showed that pharmacological inhibition of NOX4, elevated BP, increased accumulation of immune cell accumulation, and increased perivascular collagen deposition led to an accelerated vascular aging among both normotensive and hypertensive rats. Interestingly, the NOX1 inhibition did not affect the development of AH [97]. Murdoch et al. evaluated the overexpression of Nox2 and its association with AH. Thus, NOX2 contributes to the vascular remodeling and endothelial dysfunction, in addition to being involved in the pathophysiology of AH [98]. On the contrary, NOX4 promotes the protection of the vascular system in situations of increased stress induced by ischemia or angiotensin II [99]. NOX4 enhances the H_2_O_2_ production; thus, it has valuable effect on vasodilator function and BP [100].

A large body of evidence has shown that the *nuclear factor erythroid factor 2-related factor 2*(*Nrf2*) is involved in the AH pathophysiology. Tanase et al. investigated *Nrf2* in oxidative stress and its role in AH. *Nrf2* is a critical redox-sensitive transcription factor, functioning as a target nuclear receptor against oxidative stress, and it is a major component of the redox homeostasis of cells [101]. Chronic oxidative stress inhibits *Nrf2* activity and function [102]. It is worth mentioning that Farooqui and colleagues demonstrated the development of AH and renal function impairment due to an *Nrf2* inhibition in mice. The result showed that, in mice treated with a pro-oxidant—l-buthionine sulfoximine (10 mmol/L in drinking water) and an *Nrf2* inhibitor—ML385 (10 mg/kg body weight/day, intraperitoneally), oxidative stress, renal functional impairment, inflammation, and elevated BP were revealed [103]. Additionally, microRNA (miR)-140-5p exaggerates AH and oxidative stress in mouse models. Liu et al. established that downregulation of miR-140-5p reduced oxidative stress and ROS levels by activating the protein expression of *Nrf2* [104]. Furthermore, Biernacki et al. investigated changes in oxidative metabolism and apoptosis in the hearts of the hypertensive rats. The result showed that inhibition of lipolysis by fatty-acid amide hydrolase inhibitors can increase the enzymatic activity and nonenzymatic antioxidant activity in rats, whereas *Nrf2* expression is suppressed [105]. Therefore, treatments involving the upregulation of *Nrf2* expression might be promising. Nonetheless, further research is needed on the therapeutic potential of *Nrf2*.

It has become clear that inflammation, ROS, and BP elevation are significant in the pathophysiology of AH.

## 4. Conclusions

In this review, we focused on the important molecular aspects of three cardiovascular diseases: atherosclerosis, CAD, and AH. In atherosclerosis, we paid attention to the role of the oxidative processes, their pathomechanism, and the influence on the development of the disease. Important epigenetic factors, particularly those that have been highlighted in research in recent years, were also highlighted. In CAD, we focused on the endothelial dysfunction and the most important factors leading to it, as well as its new aspects. The most recent discoveries in the field of genetic background and the discovery of the role of many genes that directly or indirectly contribute to the increased risk of CAD were also considered to be of interest.

In AH, the most noteworthy aspects were the matrix metalloproteinases and the functioning of the immune system and its dysfunctions. Here, we also drew attention to the influence of oxidative stress.

These findings might shed new light on the cellular mechanisms of CVDs and prospective targets for the prevention and treatment in the near future. However, the major scientific achievements of recent years and the many new discoveries and mechanisms still require careful attention and additional studies.

## Data Availability

The data used in this article were sourced from materials mentioned in the references.
